# Automation, Monitoring, and Standardization of Cell Product Manufacturing

**DOI:** 10.3389/fbioe.2020.00811

**Published:** 2020-07-14

**Authors:** Meletios-Nikolaos Doulgkeroglou, Alessia Di Nubila, Bastian Niessing, Niels König, Robert H. Schmitt, Jackie Damen, Stephen J. Szilvassy, Wing Chang, Lynn Csontos, Sharon Louis, Patrick Kugelmeier, Vincent Ronfard, Yves Bayon, Dimitrios I. Zeugolis

**Affiliations:** ^1^Regenerative, Modular & Developmental Engineering Laboratory, National University of Ireland Galway, Galway, Ireland; ^2^Science Foundation Ireland, Centre for Research in Medical Devices, National University of Ireland Galway, Galway, Ireland; ^3^Fraunhofer Institute for Production Technology, Aachen, Germany; ^4^Production Engineering Cluster, RWTH Aachen University, Aachen, Germany; ^5^STEMCELL Technologies Inc., Vancouver, BC, Canada; ^6^STEMCELL Technologies Ltd., Cambridge, United Kingdom; ^7^Kugelmeiers Ltd., Zollikerberg, Switzerland; ^8^College System of Pharmacy, University of North Texas Health Science Center, Fort Worth, TX, United States; ^9^Cutiss AG, Zurich, Switzerland; ^10^HairClone, Manchester, United Kingdom; ^11^Medtronic – Sofradim Production, Trévoux, France

**Keywords:** cell therapy, scalability, manufacturing, monitoring, spheroid culture, biorectors

## Abstract

Although regenerative medicine products are at the forefront of scientific research, technological innovation, and clinical translation, their reproducibility and large-scale production are compromised by automation, monitoring, and standardization issues. To overcome these limitations, new technologies at software (e.g., algorithms and artificial intelligence models, combined with imaging software and machine learning techniques) and hardware (e.g., automated liquid handling, automated cell expansion bioreactor systems, automated colony-forming unit counting and characterization units, and scalable cell culture plates) level are under intense investigation. Automation, monitoring and standardization should be considered at the early stages of the developmental cycle of cell products to deliver more robust and effective therapies and treatment plans to the bedside, reducing healthcare expenditure and improving services and patient care.

## Introduction

Cell and cell-based tissue engineering products have an extraordinary clinical potential by offering unique therapeutic solutions to disease conditions without any effective treatments yet, such as non-curable cancers or non-healing or hard to heal tissues (Perez et al., 2018; Abreu et al., 2019). So far, their promises have been successfully translated only in few commercial products, primarily due to difficulties in reproducible and economical scalability, regulatory hurdles, and reimbursement issues (Morrow et al., 2017). For example, it is still challenging to translate labor-intense academic-based discoveries (automated systems often come at a prohibitive cost for academic setting and, by nature, academia is more research, as opposed to development, orientated) to automatedly manufactured industrial products. Further, the prolonged culture times required to develop a cell-based tissue engineering implantable device are associated with cell phenotypic drift and high manufacturing costs (Cigognini et al., 2013; Schrock et al., 2017; Vormittag et al., 2018). Yet again, cell therapies market size continuously raising, considering that they have the potential to transform patient care. As a fact, the global market size of cell therapies was estimated at US$ 5 billion in 2017 and it is expected to increase at a 5.34% compound annual growth rate (CAGR) until 2025 (Grand-View-Research, 2018). Although the market is shaped mainly by allogeneic therapies, autologous cell therapies are expected to rise to more than 33.3% of the total cell therapy size. Stem cell therapy market share was valued at US$ 0.8 billion in 2018 and is expected to impressively grow to US$ 11 billion by 2029 (Kanafi et al., 2013).

While there are major differences between autologous (e.g., immuno-compatible) and allogeneic (e.g., relatively readily available in large numbers) cell therapies, they share limitations in manufacturing (e.g., cell harvesting, expansion and purification; cell phenotype preservation; and development of a reproducible formulation) that may compromise the administration of a successful therapy to patients and increase costs (Aijaz et al., 2018). For example, scalable, reproducible, and biomimetic culture conditions are required to maintain cellular function during *ex vivo* culture (Liu et al., 2017; Stephenson and Grayson, 2018; Ruiz et al., 2019; Serra et al., 2019). Further, large-capacity and automated bioreactor systems have the potential to reduce batch-to-batch variability and the use of expensive highly skilled labor (Peroglio et al., 2018; Costariol et al., 2019; de Sousa Pinto et al., 2019; Hamad et al., 2019). In the case of allogeneic therapies, the aim is to scale up processes for numerous patients. In the case of autologous therapies, however, where a single patient is treated from his/her own cells, there is no need for large scale production of multiple batches with high expansion rates. Instead, manufacturers aim to culture simultaneously cells from different patients in an attempt to level up production and make it viable. An option would also be to continue culturing the cells for other patients, should appropriate consent forms be granted. Nonetheless, autologous cell therapies are still produced at small-scale, in dedicated suites, in centralized or localized manufacturing facilities at the point-of-care, which results in very expensive production costs.

In any case, both autologous and allogeneic therapies require skilled and expensive personnel, often susceptible to error, resulting in increased batch-to-batch variability, manufacturing costs and risk of contamination, which represents the biggest part of the cost of goods (COGs) for manufacturing, including tissue procurement, material acquisition, facility operation, production, storage, and shipment (Lipsitz et al., 2017). Although decentralization (Harrison et al., 2018b) and micro-factories (Harrison et al., 2018a) approaches have been proposed, automation is key for rendering these therapies more attractive, reducing the COGs, de-risking the supply chain and establishing a reliable batch-to-batch reproducibility (Hunsberger et al., 2018; Moutsatsou et al., 2019). Yet again, many questions have to be answered. For example, if the manufacturing process is scalable and suitable for automation, how can be fitted in the user requirement specifications (URS)? Regulatory considerations and ease of implementation in industrial/scalable environment are also essential. The business model should be well defined and adapted to the final product and market. The automation program should be considered as part of the full life cycle of the product, integrated into overall product development plan and its commercial manufacturing, while every potential impact of automation into the final product should be investigated. European agencies, such as the European Medicines Agency’s Innovation Task Force may assist with the development of automated processes starting with the designation of the automation, whether it should be a device or laboratory equipment. Automation challenges, coupled with lack of reliable and effective standardization, process monitoring, product reproducibility, and inadequate donor availability increase the production and reimbursement costs. It is imperative to address automation challenges in an effective way and implement process modifications with minimal disruption of the bioprocess to ensure delivery of a safe product in a commercially viable manner.

Automation offers control over a bioprocess, leading to a more accurate and faster process optimization, de-risking the supply chain, via optimized quality control, quality assurance, ultimately making the process more regulatory compliant. Although biological variations are difficult to tackle due to the complexity of the products, in-process human variation must be addressed to ensure consistent product quality. Indeed, automated pipetting, for example, can timely, accurately, repeatedly, and consistently perform liquid handling, including mixing and transferring of liquids, reducing variability within and between batches. Automation of monitoring processes (e.g., advanced algorithmic approaches, such as machine learning, coupled with image acquisition and processing) eliminates the need of subjective human judgments (e.g., cell morphology assessment, confluency assessment) further enhancing control over reproducible product development. As cell-based therapies are maturing, it is imperative to standardize and control manufacturing engineering strategies and implement robust automation and process monitoring and control for safety (above all), consistency and reproducibility purposes (Ball et al., 2018; Hunsberger et al., 2018; Pigeau et al., 2018; Moutsatsou et al., 2019). This manuscript will describe some real-life indicative examples of automation and monitoring designed to address manufacturing issues in cell-based therapies domain.

## Automating Precise Pipetting

**FIGURE 1:**
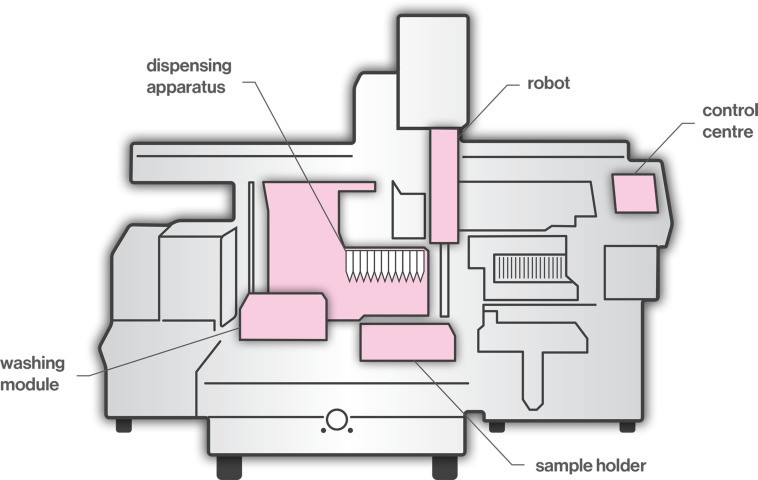
A typical liquid-handling robotic workstation.

Pipettes are laboratory tools used in the areas of chemistry, biology, and medicine, where precise, accurate and reproducible transfer of small volume of liquids is required. The accuracy of the pipetted volume can vary significantly due the quality of pipettes and tips, calibration and performance checking, environmental conditions (e.g., the temperature and density of the liquid), pipetting methods (e.g., forward or reverse), as well as the individual ability of the operator (Lippi et al., 2017). Examples of inadequate operator techniques include usage outside of pipette range, volume selection inaccuracy, fast or careless aspiration and dispensing, over-aspiration and barrel contamination. Considering the potentially high levels of user-dependent inaccuracy, in recent years, the use of automated pipetting systems has increased significantly to meet the need for high accuracy and high throughput in biomedical laboratories. Robots work without fatigue, perform consistently, increase the production and ensure accuracy and precision. A typical liquid-handling robotic workstation (Figure 1) consists of a control center, dispensing apparatus, robots, washing modules, and sensors (Kong et al., 2012). The robot, coordinated by the control center, moves between the dispensing part and the washing station. Dispensing tools include dispensing heads, actuators, and substrates. The dispensing head expels liquid samples on the substrates for further processing. The washing station cleans the dispensing head to lengthen its life and to ensure the integrity of the sample. Sensors monitor the status of the dispensing component to ensure that feedback control can be performed by the control center. One of the main challenges regarding automated pipetting systems is the viscous material handling. The key factor is the distance between the dispensing needle tip and the base of the well (Peddi et al., 2007; Yaxin et al., 2011). If the distance is too great, the sample from the needle forms a continuous cylinder and is not delivered to the well, whereas, if the distance is too short, the sample remains attached to the needle. In order to choose an adequate distance, it is necessary to consider relevant parameters, such as needle size, syringe volume, pumping temperature, flow speed, and viscosity grade. Moreover, dusty and viscous materials, air bubbles or the accumulation of liquid debris may cause clogging in tubes, valves and dispensing heads; thus, clogging detection is required (Kong et al., 2012).

Automated precise pipetting plays a central role in cell culture automation. Automated cell culture systems enable large-scale production of cells and enhance technical precision, reproducibility and efficiency (Konagaya et al., 2015). Monitoring the flow rate, for example, during media change, is an important operation to ensure that shear forces on cells are contained (Ly et al., 2013). A fine-tuning of the pipetting settings could decrease the shear stress, but very slow aspirating steps are associated with a long duration of the process. However, a benefit of automation should be a reduced process time compared to manual operations (Lehmann et al., 2016). A prerequisite for the successful implementation of automated procedures in cell culture experiments is a complete and adequate validation, during which automated pipetting systems are directly compared to manual pipetting, conducted by an experienced laboratory technician. In a previously published case study (Rothmiller et al., 2020), toxicity studies in HaCaT cells were conducted using two epMotion^®^ automated pipetting systems (Eppendorf, Germany), which were validated / contrasted against an experienced. Validation analysis revealed that automated seeding was faster and more precise than manual seeding, with a significantly lower variability and equivalent intraday variability. Collectively, automated pipetting, if it is not already, should become an industry standard for accurate, reproducible and cost-effective development of cell-based products.

## Automation and Screening

**FIGURE 2:**
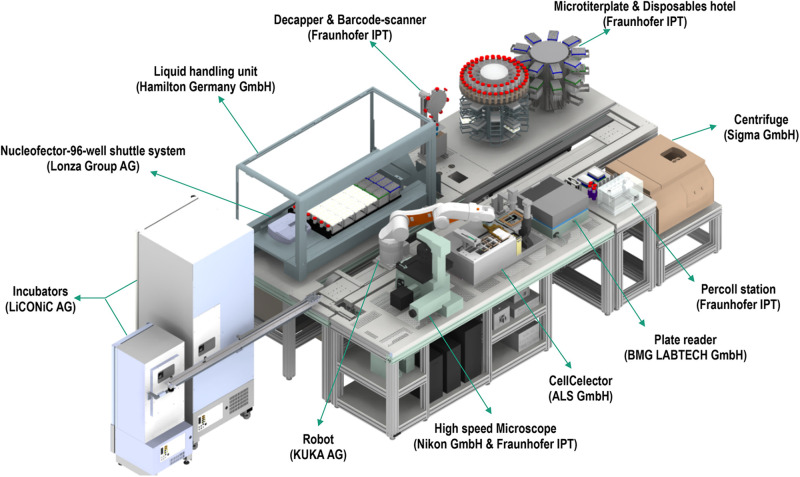
The StemCellFactory, an automated system for reprogramming and expansion of iPSCs.

In an increasing and demanding tissue engineering market, advanced automation and screening for quality control are essential for sustainability. In this direction, recent commercial efforts have made available automated systems for cell manufacturing (e.g., CliniMACS Prodigy^®^, Miltenyi Biotec; Sefia S-2000, GE Healthcare, Life Sciences). For industrialization and manufacturing, quality control requires well-characterized, fully reproducible and safe products to ensure delivery of the expected medical benefits. Considering that cell morphology is indicative of phenotype, effective monitoring of cells’ and cell clusters’ morphology are prerequisites for standardization and homogenous product delivery (Maddah et al., 2014; Boutros et al., 2015; Nagasaka et al., 2017). Microscopic observations are the routine method used for the assessment of cell culture. The need for automated and fast evaluation has led to the development of machine learning algorithms and artificial intelligence, able to assess morphological and functional properties of cell culture. The principle of machine learning includes the development of algorithms that are being trained by data input, thus improving their intrinsic processes and providing more accurate outputs. Since many single or populational characteristics would indicate the suitability of cells for further experimentation, it is imperative that techniques can fast and accurately process large volume of data (e.g., images). Indeed, image processing machine learning techniques have been successfully implemented and validated in oncological studies to predict specific function based on gene phenotype similarities (Sailem et al., 2020), in predicting cell growth per passage from batches obtained from donors varying in age (Mehrian et al., 2020) or phenotypically and structurally evaluating different cell types (Logan et al., 2016; Van Valen et al., 2016; Wakui et al., 2017; Buskermolen et al., 2018; Radio and Frank, 2018; Lam et al., 2019; Orita et al., 2019). Following successful implementation of machine learning for cell morphology analysis, automation on the level of cell production and screening is the next vital step, which systems, such as the StemCellFactory, aspire to achieve. This system automates reprogramming and expansion of induced pluripotent stem cells (iPSCs) for disease modeling and drug screening (Jung et al., 2018). The system is comprised of various devices (Figure 2), which are functionally joined and integrated into a central control system orchestrating the process execution and data handling.

**FIGURE 3:**
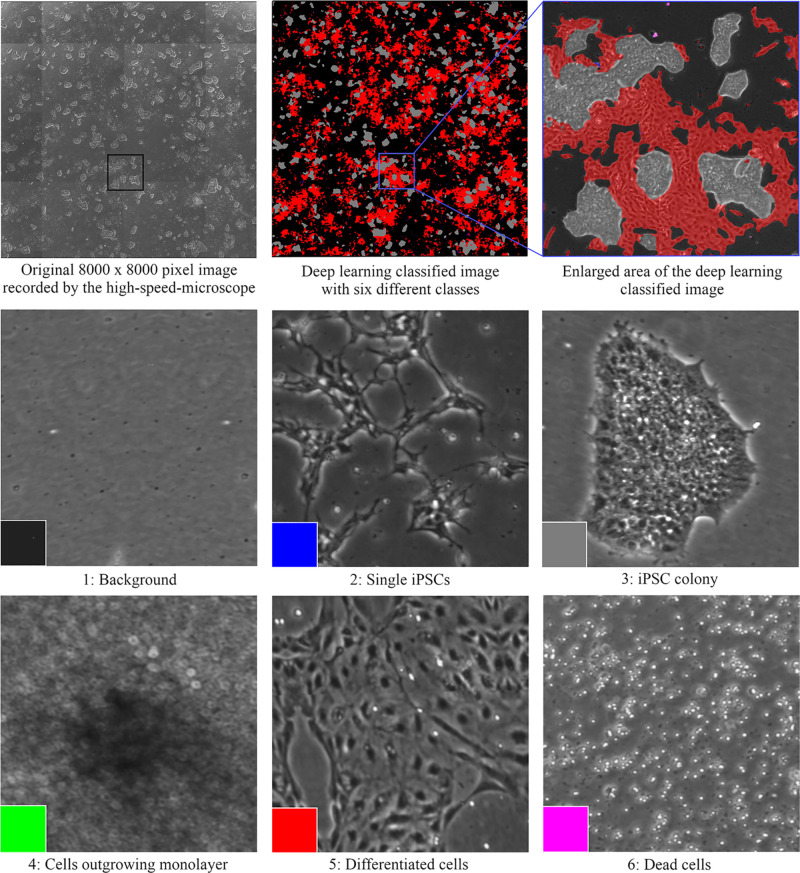
Cell classification with the deep learning algorithms that are color-indicated (background is depicted in black, single iPSCs in blue, iPSC colonies in gray, cells in 3D structure in green, differentiated cells in red, and dead cells in purple).

Each device has its local software agent, which serves as middleware interface and abstracts the hardware heterogeneity by offering data and functionality in a service-oriented way to the control unit. Local information and functionality from the individual device are processed in the middleware up to the higher-level of the control system, such that the user only operates one software with control over the complete system. In order to expand and monitor the iPSCs, the system is equipped with an automated microscope to assess their morphological structure and confluency level. The control system utilizes data handling and flexible process control to perform the tasks. For example, the user can input a confluence level that will lead to cell splitting or media change. Due to the high amount of data generated (20 GB per media transfer protocol) and the needed high computational power for evaluation, deep learning algorithms are used. These algorithms classify an image into six different classes that are color-indicated (Figure 3). Automation can also be achieved in genome editing or reprogramming during cell culture steps. To this end, for the automated detection of iPSC colonies, the CellCelector system is implemented, which allows automated picking of clones for subsequent clonal expansion on the StemCellFactory. Even more, the Nucleofector device allows automated genome editing. So far, the StemCellFactory has been used for the automated reprogramming of human dermal fibroblasts, clonal selection and expansion of primary iPSC clones and scaled enzyme-free sub-cultivation of iPSC lines. To summarize, the StemCellFactory can provide reproducible results, growth behavior monitoring, high throughput through parallelization. These automated platform and novel software tools address the technological challenges for automation of complex stem cell culture processes and are expected to meet the challenges of the increasing demand for patient-derived iPSCs and their derivatives.

## Automated Classification and Quantitation of Colonies of Blood Cells

**FIGURE 4:**
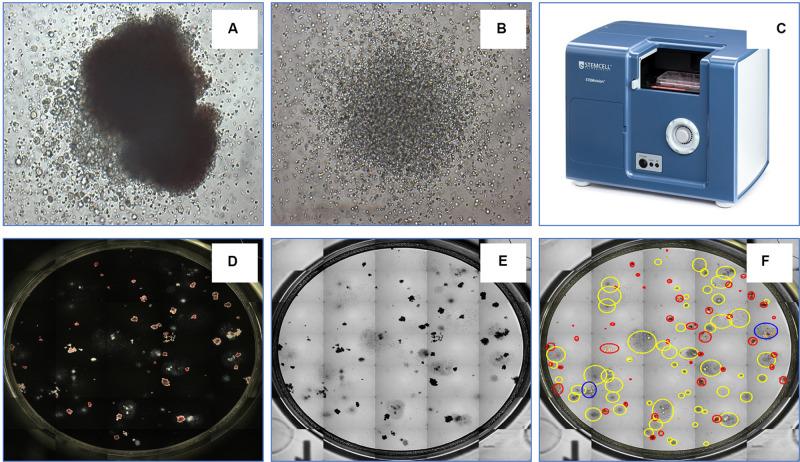
An example of a CFU-GEMM **(A)** and CFU-GM **(B)** in a typical 14 day CFU assay from bone marrow-derived hematopoietic stem cells. The automated colony-forming unit counting and characterization instrument, STEMvision^TM^ **(C)**. STEMvision records both dark-field **(D)** and bright-field **(E)** images of CFUs and utilizes state-of-art software to identify colonies. **(F)** Yellow circles represent CFU-GM; blue circles represent CFU-GEMM; and red circles represent BFU-E.

The transplantation of hematopoietic stem and progenitor cells (HSPC) from human bone marrow (BM), adult mobilized peripheral blood (MPB) or umbilical cord blood (CB) has for more than 50 years been employed as an effective treatment for a variety of blood disorders and malignancies (Juric et al., 2016; Takami, 2018; DeFilipp et al., 2019). An important approach to assess their potency and predict the likelihood of robust engraftment is to determine the number and quality of lineage-specific progenitor cells and multipotent stem cells among the cells to be transplanted. Many studies have shown that the number of HSPCs is directly correlated with engraftment outcomes (Prasad et al., 2008; Page et al., 2011). Several criteria are commonly used to establish graft potency and quality, including the total number of viable nucleated cells, the number of cells expressing the CD34 antigen and the number of cells able to produce discrete colonies of mature blood cells upon culture in semi-solid growth media. Hematopoietic cells with the latter capability form colony-forming units (CFU) and the CFU assay is the current gold standard for determining the number of functional HSPCs. Hematopoietic progenitor cells can differentiate into several blood cell lineages in the CFU assay. Depending on the growth factors present in the culture medium, the assay can identify (a) erythroid progenitor cells that produce either very small or medium to large colonies comprised of pure red blood cells [i.e., colony-forming units-erythroid (CFU-E) and burst-forming units-erythroid (BFU-E), respectively]; (b) uni- or bi- potent myeloid progenitor cells [i.e., colony-forming units granulocyte (CFU-G), colony-forming units macrophage (CFU-M), and colony-forming units granulocyte-macrophage (CFU-GM)]; or (c) multipotent progenitor cells that generate large colonies comprised of all four major non-lymphoid cell types [colony-forming units granulocyte, erythrocyte, macrophage, megakaryocyte (CFU-GEMM)]. Shown in Figure 4 are examples of colonies derived from CFU-GEMM and CFU-GM with each exhibiting distinct morphological features, most notably different size and cellular composition. These colonies produced by CFU *in vitro* are usually counted manually using an inverted microscope by trained personnel with (ideally) extensive experience, but who nevertheless must often make difficult judgements on the boundaries and composition of the discrete colonies that they observe. For example, colonies produced by BFU-E or CFU-GEMM share overlapping characteristics that pose challenges to colony classification. This can contribute to a degree of inter-individual variability in CFU assay scoring accuracy, typically between 10 and >100% depending on the colony sub-type (Pamphilon et al., 2013). In addition, manual counting and characterization of CFU colonies is labor intensive. Thus, an automated solution would increase both the speed and accuracy and facilitate standardization in performing the CFU assay.

Toward these objectives, STEMCELL Technologies Inc. developed STEMvision^TM^ (Figure 4), a bench-top instrument designed specifically for imaging, classifying and counting hematopoietic colonies produced by human or mouse progenitor cells in the CFU assay. The instrument separately counts and identifies colonies generated by CFU-E, BFU-E, CFU-GM, or CFU-GEMM that develop in the conventional 14-day CFU assay performed using MethoCult^TM^, a line of semi-solid methylcellulose-based culture media supplemented with combinations of hematopoietic growth factors that stimulate the survival, proliferation and differentiation of all sub-types of CFUs. STEMvision^TM^ eliminates the inter- and intra-individual and laboratory variations associated with manual colony counting by using sophisticated image acquisition and analysis software to identify and classify hematopoietic colonies. The morphological criteria that facilitate classification of the different sub-types of CFUs are applied consistently, facilitating standardization of the CFU assay to ensure accuracy and reproducible results. All of the common and particularly challenging phenomena encountered when counting CFU assays are addressed. For example, colonies can occasionally present with multiple foci or clusters, which some individuals may consider to be separate colonies thus erroneously skewing the total count to higher CFU numbers that in turn may lead to an overestimation of HSPC graft potency. Conversely, colonies at the edge of the culture dishes may be missed in the shadow produced by the meniscus of the MethoCult^TM^ medium, leading to under-counting of CFUs and under-estimation of graft potential. By performing the assay in SmartDish^TM^ culture plates that prevent meniscus formation and employing standardized imaging and analysis software that are specifically developed and validated for counting all of the different types of colonies produced by CFU from BM, MPB or CB, use of this platform results in significantly greater accuracy and less variability in colony counts. Improved colony characterization is also accomplished by analyzing colony features from both dark-field (i.e., black and white) and bright-field (i.e., color) images (Figure 4) to improve automated decision-making. Following analysis with STEMvision^TM^, data can be visualized in a pdf report format that can be pre-filled with information, such as donor ID, sample ID, number of cells plated and additional qualifiers defined by the user. The report is automatically generated and results are expressed as CFU frequencies with digital images of the analyzed cultures available for manual review and long-term archiving. The plate and sample ID are linked to each image for traceability and time stamped.

The development of new gene-editing tools such as CRISPR/Cas9 technologies has opened up new avenues for gene therapy approaches to blood disorders and researchers are vigorously testing and optimizing new protocols for correcting genetic defects in HSCs (Dever et al., 2016; Naldini, 2019). Given the current guidelines for quality and process control for all types of manipulated HSCs, advancement of HSPC-based cellular therapies will certainly depend increasingly on the use of standardized potency assays, such as the CFU assay, especially when these cells are modified through CRISPR/Cas9 targeting prior to transplantation. Current guidelines set out by the FDA specify that frozen CB units must be tested not only for cell viability, but also for potency since cryopreservation and thawing are often associated with reduced growth and differentiation capacity of HSPCs (Watts and Linch, 2016). Several investigators have validated STEMvision^TM^ for standardizing the CFU assay within and across labs (Velier et al., 2019) and it is clear that STEMvision^TM^ provides an effective automated approach to classify and count CFUs during the evaluation of hematopoietic stem cell viability and potency.

## 3D Cell Culture Systems: The Case of Pancreatic Islet Cell Clusters for Type I Diabetes

Diabetes affects globally >382 million individuals with expected increase almost to 600 million in the next 15 years (Guariguata et al., 2014; Cho et al., 2018). There are different types of diabetes, of which type 1 diabetes has gained more attention due to its autoimmune nature. Type 1 diabetes is associated with malfunction of the pancreatic islets and more specifically with the destruction of the insulin-producing cells (beta cells), which reside inside the islets. Destruction of beta cells leads to insufficiency of insulin production from the body, leading to inability of glucose entering the cells, which leads to elevated level of sugars into the bloodstream (Fu et al., 2013; Kettunen and Tuomi, 2020). Moreover, diabetes is the main cause for kidney disease with a correlation of 25% of diabetic people resulting in kidney failure (Guariguata et al., 2014; Marshall, 2014). To be safe and effective, islet cell transplantation needs size standardization, which would lead to much higher cell survival due to better oxygenation of the islet (Papas et al., 2019). In addition, limited availability, immuno-rejection and procedure issues should be addressed to alleviate islet cell loss and to improve engraftment outcomes (Shapiro et al., 2017; Gamble et al., 2018).

*In vitro* cell culture platforms have the potential to standardize islets (Hilderink et al., 2015; Ichihara et al., 2016; Vlahos et al., 2019) and also provide an environment to prepare autologous or allogeneic stem cell therapies for diabetes (Lilly et al., 2016; Cierpka-Kmiec et al., 2019; Kumar et al., 2019). In recent years three dimensional spheroid culture systems have emerged that simulate more effectively the physiological tissue microenvironment due to the cell-cell and cell-ECM contact and interaction (Mitchell, 2017; Langhans, 2018). Cells spheroids have shown improved osteogenic, adipogenic and chondrogenic potential over conventional culture systems (Yoon et al., 2012; Yamaguchi et al., 2014; Cesarz and Tamama, 2016; Miyamoto et al., 2017; Moritani et al., 2018; Tsai et al., 2019), improved vascularization in ischemic tissue (Bhang et al., 2012) and constitute the first choice in cancer models and evaluation of anti-cancer drugs (Chatzinikolaidou, 2016; Zanoni et al., 2016; Rodrigues et al., 2018). Various scaffold-free [e.g., seeding cells in a porous microwell agarose microchip (Colle et al., 2020), seeding cells in 3D printed well inserts (Boyer et al., 2018) or the hanging drop method (Kapur et al., 2012)] and scaffold-based [e.g., natural or synthetic hydrogels are used as substrates for spheroids growth (Murphy et al., 2014; Chang et al., 2018; Lee et al., 2018; Kim et al., 2019)] have been described in the literature. Regarding scalability, of significant importance are recent studies that describe scaffold-free cell spheroids production using the hanging drop method performed by a robotic device (Gutzweiler et al., 2017) and a robotic automated droplet microfluidic platform (Langer and Joensson, 2020). We should also mention that automated production of cell spheroids in outer space has also been documented (Pietsch et al., 2017).

In diabetes field, cell spheroids can provide an inducive environment for islet differentiation from stem cells, upregulate stemness factors and allow production of angiogenic and non-thrombogenic therapies (Moritz et al., 2002; Oh et al., 2018; Lo et al., 2019). Early data of islets/mesenchymal stem cell co-cultures in spheroids demonstrated improved islet long term viability, but not function (Rawal et al., 2017). Recent data however study demonstrated that incorporation of human amniotic epithelial cells into islet organoids to markedly enhance engraftment, viability and graft function in a mouse type 1 diabetes model (Lebreton et al., 2019). Although the potential of spheroids in regenerative medicine has already been demonstrated in preclinical models for most organ systems (Hagemann et al., 2017; Petrenko et al., 2017; Polonchuk et al., 2017; Ong et al., 2018), their slow clinical translation may be attributed to variable cluster size, which affects cell response (Moritz et al., 2002; Van Hoof et al., 2011; Anitha et al., 2020). Thus, spheroid production must be standardized to bridge the gap between preclinical testing and clinical translation.

**FIGURE 5:**
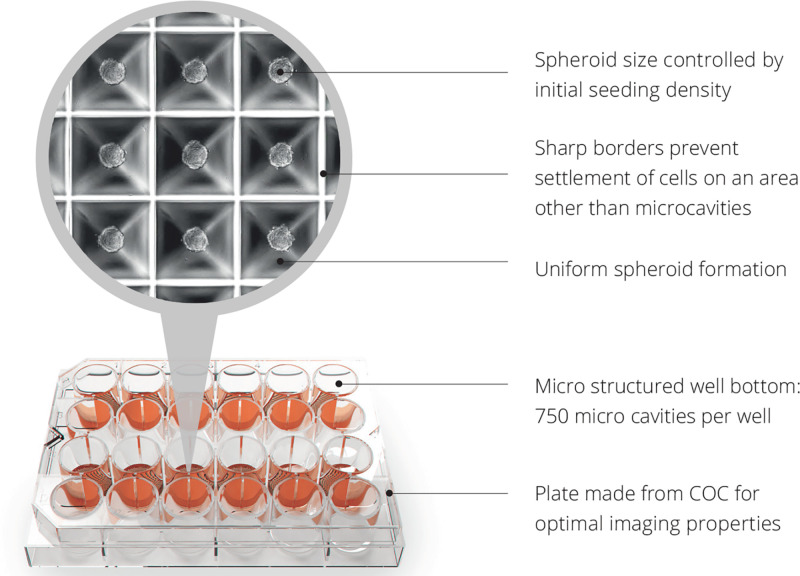
Spheroid development with Sphericalplate 5D. COC, cycloolefin copolymer.

The link between islet transplantation and regenerative medicine is that islet transplantation is the only spheroid/cluster/organoid transplantation in the world that is being performed in a routine clinical fashion for 20 years (Bottino et al., 2018). Therefore, the clinical experience of islet transplantation can be taken as a ‘blueprint’ for future cell therapies with spheroids. The format and thus handling, challenges and principles are literally the same. Having said that, even in classical islet transplantation, the formed cell clusters are not flawless, mainly due to anoxia occurring in the center of large clusters due to the high diffusion distance (Brandhorst et al., 2016). After transplantation, the only oxygen supply path is through diffusion whereas they remain in hypoxic condition in the portal system (Moritz et al., 2002). This is the reason why currently 80–90% of the transplanted islet cells are not surviving the first days of transplantation (Suszynski et al., 2016). Oxygen consumption is also directly correlated to the insulin production (Porterfield et al., 2000) and cluster size (Labuschagne et al., 2019). Indeed, *in vitro* and clinical data in patients suffering from type 1 diabetes have shown in large clusters less insulin-expressing cells both in normoxic and hypoxic conditions and the larger islets were significantly reduced in size under hypoxia (Lehmann et al., 2007). The Sphericalplate 5D is an example of a cell culture platform that can produce regular cell clusters in the desired numbers and quality for improved clinical islet transplantation and future applications with spheroids (Zuppinger, 2019; Schulze-Tanzil et al., 2020). The shape of the Sphericalplate 5D platform allows size standardization and also correct stem cell communication within the formed spheroids by recreating the physiological niche environment in thousands of microwells (Figure 5). Regarding scalability and automation, the Sphericalplate 5D platform fulfils necessary first principles of clinical cell transplantation, such as reproducibility, medium change capacity and optimized mechanobiology for every single spheroid (Kugelmeier et al., 2010). So far, diverse populations of cells (e.g., islets, embryonic stem cells, iPSCs, BM stem cells, prostate cancer cells, hepatocytes) have been successfully expanded in this platform (Schmidhauser et al., 2019) and the first clinical trial is planned for 2021 to improve current islet cell transplantation by standardizing spheroid/cluster size and consequently cell survival in the Sphericalplate 5D. More such scalable and, hopefully, effective technologies will enable the development of functional and affordable cell therapies.

## Conclusion

Cell-based therapies have the potential to offer an effective treatment to still uncurable disease conditions. Their broad commercialization has been jeopardized by limitations (e.g., scaling up and automating labor-intense academic discoveries, high manufacturing costs and variation between batches) in large scale automated manufacturing. Herein, we discussed examples in the field of cell manufacturing automation, monitoring and standardization. Such successful examples of automated and controlled cell product manufacturing and monitoring should inspire the development of cost-effective cell products for the benefit of patients still suffering from uncurable diseases.

## Author Contributions

All authors listed have made a substantial, direct and intellectual contribution to the work, and approved it for publication.

## Conflict of Interest

JD, SS, WC, LC, and SL are employees of STEMCELL Technologies. PK is Director of Science and Founder of Kugelmeiers AG. VR is Chief Innovative Officer of Cutiss AG and Chief Scientific Officer of HairClone Ltd. YB is an employee of Sofradim Production, A Medtronic Company. The remaining authors declare that the research was conducted in the absence of any commercial or financial relationships that could be construed as a potential conflict of interest.
